# Using Decision Forest to Classify Prostate Cancer Samples on the Basis of SELDI-TOF MS Data: Assessing Chance Correlation and Prediction Confidence

**DOI:** 10.1289/txg.7109

**Published:** 2004-08-05

**Authors:** Weida Tong, Qian Xie, Huixiao Hong, Hong Fang, Leming Shi, Roger Perkins, Emanuel F. Petricoin

**Affiliations:** ^1^Center for Toxicoinformatics, Division of Biometry and Risk Assessment, and; ^2^Bioinformatics Group, National Center for Toxicological Research, Jefferson, Arkansas, USA; Center for Biologics Evaluation and Research, U.S. Food and Drug Administration, Bethesda, Maryland, USA

**Keywords:** bioinformatics, chance correlation, class prediction, classification, decision forest, prediction confidence, prostate cancer, proteomics, SELDI-TOF

## Abstract

Class prediction using “omics” data is playing an increasing role in toxicogenomics, diagnosis/prognosis, and risk assessment. These data are usually noisy and represented by relatively few samples and a very large number of predictor variables (e.g., genes of DNA microarray data or *m/z* peaks of mass spectrometry data). These characteristics manifest the importance of assessing potential random correlation and overfitting of noise for a classification model based on omics data. We present a novel classification method, decision forest (DF), for class prediction using omics data. DF combines the results of multiple heterogeneous but comparable decision tree (DT) models to produce a consensus prediction. The method is less prone to overfitting of noise and chance correlation. A DF model was developed to predict presence of prostate cancer using a proteomic data set generated from surface-enhanced laser deposition/ionization time-of-flight mass spectrometry (SELDI-TOF MS). The degree of chance correlation and prediction confidence of the model was rigorously assessed by extensive cross-validation and randomization testing. Comparison of model prediction with imposed random correlation demonstrated biologic relevance of the model and the reduction of overfitting in DF. Furthermore, two confidence levels (high and low confidences) were assigned to each prediction, where most misclassifications were associated with the low-confidence region. For the high-confidence prediction, the model achieved 99.2% sensitivity and 98.2% specificity. The model also identified a list of significant peaks that could be useful for biomarker identification. DF should be equally applicable to other omics data such as gene expression data or metabolomic data. The DF algorithm is available upon request.

Recent technologic advances in the fields of “omics,” including toxicogenomics, hold great promise for the understanding of the molecular basis of health and disease, and toxicity. Prospective further advances could significantly enhance our capability to study toxicology and improve clinical protocols for early detection of various types of cancer, disease states, and treatment outcomes. Classification methods, because of their power to unravel patterns in biologically complex data, have become one of the most important bioinformatics approaches investigated for use with omics data. Classification uses supervised learning techniques (Tong et al. 2003b) to fit the samples into the predefined categories based on patterns of omics profiles or predictor variables (e.g., gene expressions in DNA microarray). The fitted model is then validated using either a cross-validation method or an external test set. Once validated, the model could be used for prediction of unknown samples.

A number of classification methods have been applied to microarray gene expression data (Ben-Dor et al. 2000; Simon et al. 2003; Slonim 2002), including artificial neural networks (Khan et al. 2001), *K*-nearest neighbor (Olshen and Jain 2002), Decision Tree (DT; Zhang et al. 2001), and support vector machines (SVMs; Brown et al. 2000). Some of the same methods have been applied similarly to proteomic data generated from surface-enhanced laser deposition/ionization time-of-flight mass spectrometry (SELDI-TOF MS) for molecular diagnostics (Adam et al. 2002; Ball et al. 2002). For example, Petricoin et al. (2002a, 2002b) developed classification models for early detection of ovarian and prostate cancers (PCAs) on the basis of SELDI-TOF MS data using a genetic algorithm–based SVM.

Omics data present challenges for most classification methods because *a*) the number of predictor variables normally far exceeds the sample size and *b*) most data are unfortunately very noisy. Consequently, optimizing a classification model inherently risks overfitting the noise, a result that is difficult to overcome for most classification methods (Slonim 2002). Furthermore, many existing classification methods require predetermination of a set of predictor variables, thereby introducing additional complexity and bias that could adversely affect both model fitting and validation (Ambroise and McLachlan 2002).

In this article a novel classification method, Decision Forest (DF), is proposed for developing classification models using omics data. A DF model is developed by combining multiple distinct but comparable DT models to achieve a more robust and better prediction (Tong et al. 2003a). DF does not require predetermination of predictor variables before model development and is less prone to overfitting of noise. Developing a statistically sound model that fits the data is straightforward with most classification methods, but assuring that the model can accurately classify unknown samples with a known degree of certainty poses a significant challenge. In DF, an extensive cross-validation and randomization testing procedure was implemented, which provides two critical measures to assess a fitted model’s ability to predict unknown samples, the confidence level of predictions and the degree of chance correlation. DF is demonstrated in an application to distinguish PCA samples from normal samples on the basis of a SELDI-TOF MS data set. The results indicate that the reported DF model could be useful for early detection of PCA.

## Materials and Methods

### Proteomics Data Set

A proteomic data set reported by Adam et al. (2002) is used in this study. The data set consists of SELDI-TOF MS spectra for 326 samples, which is generated using the IMAC-3 chip (Ciphergen Biosystems, Inc., Fremont, CA). Of 326 serum samples used, 167 samples were from the PCA patients, 77 from the patients with benign prostatic hyperplasia (BPH), and 82 from healthy individuals. The samples were subsequently divided into two classes for this study, cancer samples (167 PCA samples) versus noncancer samples (159 samples including both BPH and healthy individuals) (Qu et al. 2002). Each sample was characterized by 779 peaks of a spectrum. These peaks were determined in the mass range of 2,000–40,000 Da and provided by the original authors (Adam et al. 2002) for this study. All these peaks were used as predictor variables without preselection to develop the DF model.

### Decision Tree

A DT model was developed using a variant of the classification and regression tree (CART) method (Breiman et al. 1995), which consists of two steps—tree construction and tree pruning (Clark and Pregibon 1997). In the tree construction process the algorithm identifies the best predictor variables that divide the sample in the parent node into two child nodes. The split maximizes the homogeneity of the sample population in each child node (e.g., one node is dominated by the cancer samples, and the other is populated with the noncancer samples). Then, the child nodes become parent nodes for further splits, and splitting continues until samples in each node are either in one classification category or cannot be split further to improve the quality of the DT model. To avoid overfitting the training data, the tree is then cut down to a desired size using tree cost-complexity pruning (Clark and Pregibon 1997). In the end of the process, each terminal node contains a certain percentage of cancer samples. This percentage specifies the probability of a sample to be the cancer sample. In this study the cutoff 0.5 was used to distinguish cancer samples from noncancer samples. If a terminal node contains the proportion of cancer sample (*p*) > 50% (i.e., *p* > 0.5), all the samples in this terminal are designated as cancer samples and *p* is the probability value assigned to the entire sample in this terminal node. Similarly, samples are noncancer if the probability is < 0.5.

### Decision Forest

DF is a consensus modeling technique, where the results of multiple DT models are combined to produce a more accurate prediction than any of the individual independent DT models. Because combining several identical DT models produces no gain, the rationale behind DF is to develop multiple DT models that are heterogeneous with comparable quality. “Heterogeneity” emphasizes each DT model’s unique contribution to the combined prediction, which is accomplished by developing each DT model based on a distinct set of predictor variables. “Comparable quality” ensures each DT model’s equal weight in combining prediction, which requires each DT model having similar accuracy of prediction. Thus, the development of a DF model consists of three steps (Tong et al. 2003a): *a*) develop a DT model, *b*) develop the next DT model based on only the predictor variables that are not used in the previous DT model(s), and *c*) repeat the first two steps until no additional DT models can be developed. In this process the misclassification rate for each DT model is controlled at a fixed level (3–5%) to ensure the comparable quality of individual DT models. The same classification call in DT is used for determining a sample’s classification based on the mean probability value of all DT models used in DF.

### Randomization Test for Chance Correlation

Because proteomic data usually contain a large number of predictor variables with a relatively small number of samples, it is possible that the patterns identified by a classification model could be simply due to chance. Thus, we used a randomization testing to assess the degree of chance correlation. In this method the predefined classification of the samples was randomly scrambled to generate 2,000 pseudo-data sets (Good 1994). The DF models were developed for each pseudo-data set, and the results were then compared with the DF model from the real data set to determine the degree of chance correlation.

### Model Validation

A common approach for assessing the predictivity of a classification model is to randomly split the available samples into a training set and a test set. The predictivity of a fitted model using all the samples is estimated based on the prediction accuracy for the test set. Arguably, the cross-validation method could be considered as an extension of this external validation procedure and might offer an unbiased way to assess the predictivity of a model from a statistical point of view (Hawkins et al. 2003). In this procedure a fraction of samples in the data set are excluded and then predicted by the model produced using the remaining samples. When each sample is left out one at a time, and the process repeated for each sample, this is known as leave-one-out cross-validation (LOO). If the data set is randomly divided into *n* groups with approximately equal numbers of samples, and the process is carried out for each group, the procedure is called leave-*n*-out cross-validation (LNO). Because LOO gives a minimal perturbation to the data set and therefore might not detect overfitting of a model, the leave-10-out cross-validation (L10O) is commonly used for classification models.

It is important to point out that the LNO results vary for each run because the partition of the data set is changing in a random manner (except for the LOO procedure). The variation increases as the number of left-out samples increases (i.e., *n* decreases with *n* > 1). Care must be taken when interpreting the results derived from only one pass through an LNO process, which could lead to a conclusion that might not represent the true predictivity of the fitted model due to chance. Rather, the mean of many passes through the LNO process should well approximate the predictivity of the fitted model. In this study an extensive L10O procedure was implemented in DF, where the L10O process was repeated 2,000 times using randomly divided data sets in each run. The choice of 2,000 runs is based on our previous experience of where reliable statistics can be reached (Tong et al. 2003a). In this validation process a total of 20,000 pairs of training and test sets were generated, and each sample was predicted by 2,000 different models. The results derived from this process provide an unbiased statistic for evaluating the predictivity of a fitted model.

## Results

**Figure 1:**
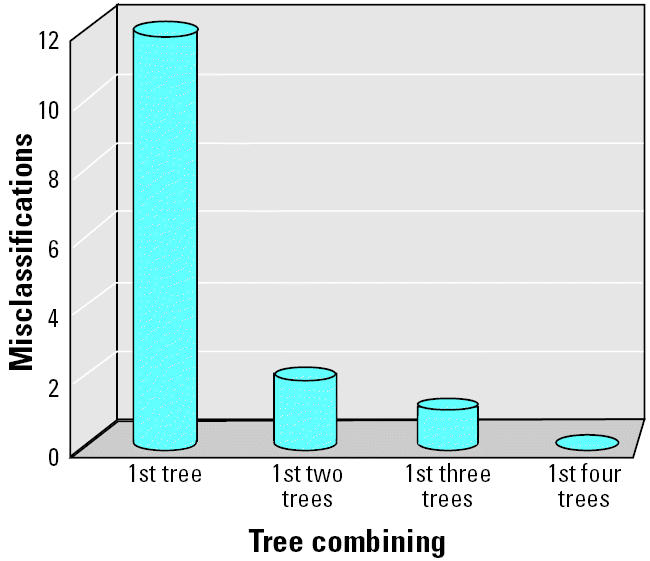
Plot of misclassifications versus the number of DT models to be combined in DF.

DF was applied to the proteomic data set for distinguishing cancer from noncancer. The fitted DF model for the data set contains four DT models, each of them having the comparable misclassifications ranging from 12 to 14 (i.e., 3.7–4.3% error rate; Table 1). The misclassification is significantly reduced as the number of DT models to be combined increases to form a DF model (Figure 1). The four-tree DF model gave 100% classification accuracy. However, it is important to note that a statistically sound fitted model provides limited indication of whether the identified pattern is biologically relevant or is solely due to chance. Neither does such a fitting result provide validation of the model’s capability for predicting unknown samples that were not included in the training set used for model development. It is important to carry out a rigorous validation procedure to determine the fitted model with respect to the degree of chance correlation and the level of confidence for predicting unknown samples.

### Assessment of Chance Correlation

**Figure 2:**
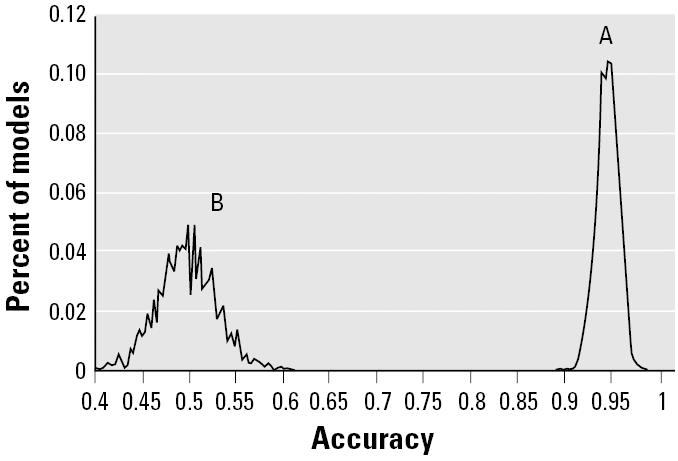
Prediction distribution in the 2,000-L10O process: real data set (*A*) and 2,000 pseudo-data set (*B*) generated from a randomization test.

We compared the predictive accuracy for the left-out samples in the 2,000 L10O runs of the real data set (total of 20,000 pairs of training and test sets) with those derived from the L10O run for each of the 2,000 pseudo-data sets (total of 20,000 pairs of training and test sets). The distributions of the prediction accuracy of every pair for both real and pseudo-data sets are plotted in Figure 2. The distribution of prediction accuracy of the real data set centers around 95%, whereas the pseudo-data sets are near 50%. The real data set has a much narrower distribution compared with the pseudo-data sets, indicating that the training models generated from the L10O procedure for the real data set give consistent and high prediction accuracy with their corresponding test sets. In contrast the prediction results of each pair of training and test sets in the L10O process for the pseudo-data sets varied widely, implying a large variability of signal:noise ratio among these training models. Importantly, there is no overlap between two distributions, indicating that a statistically and biologically relevant DF model could be developed using the real data set.

### Assessment of Prediction Confidence

**Figure 3:**
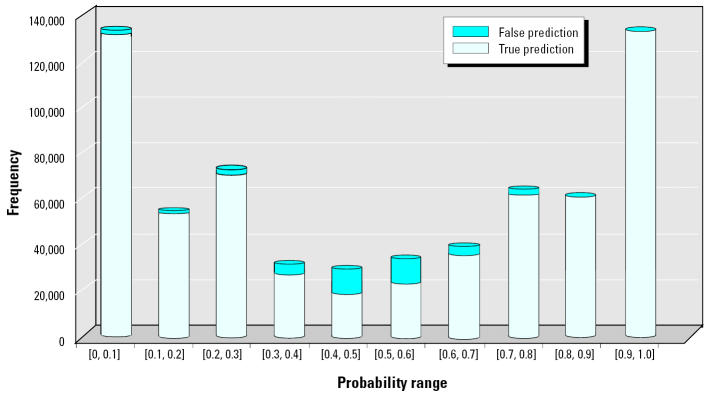
Distribution of true/false predictions for the left-out samples over 10 equal-probability bins in the 2,000-L10O process.

DF assigned a probability value for each prediction, where samples with the probability value ≥ 0.5 were designated as cancer samples, whereas others were designated as normal samples. Figure 3 provides two sets of information derived from the 2,000 L10O runs over 10 equal probability intervals between 0 and 1: *a*) the number of left-out samples predicted in each bin and *b*) the misclassification rate in each bin. Analysis shows that the 0.7–1.0 interval has a concordance of 99.2% for the cancer samples (0.8% false positives), whereas the 0.0–0.3 interval has a concordance of 98.2% for the noncancer sample (1.8% false negatives). These two probability ranges accounted for 79.7% of all left-out samples. The vast majority of misclassifications occur in the 0.3–0.7 probability range, where the average prediction accuracy was only 78.9% but which, fortunately, accounted for only 20.3% of total of left-out samples. Therefore, we defined both the predicted probability ranges of 0.0–0.3 and 0.7–1.0 as the high-confidence (HC) region, whereas the predicted probability range of 0.3–0.7 was considered the low-confidence (LC) region.

### Comparison of DF with DT

Table 2 summarizes the statistical results of the 2,000 L10O runs for both DF and DT. Overall, the DF model increases prediction accuracy by about 5% compared with the DT model, from 89.4 to 94.7%. In the HC region, the DF model increases prediction accuracy compared with the DT model by 8% from 90.7 to 98.7%, compared with 15% from 63.8 to 78.9% in the LC region.

### Biomarker Identification

In addition to development a predictive model for proteomic diagnostics, identification of potential biomarkers is another important use of the SELDI-TOF MS technology (Diamandis 2003). Each DT model in DF determines a sample’s classification through a series of rules based on selection of predictor variables. Thus, it is expected that the DF-selected variables could be useful as a starting point for biomarker identification.

There were two lists of model-selected variables derived from DF, one used in fitting (the fitting-variable list; Table 1) and the other used by at least one of the models in the 2,000-L10O process (the L10O-variable list). The L10O-variable list contained 323 unique variables, which actually included all variables in the fitting-variable list. Given that the sample population is different among the models in the 2,000 L10O runs, the number of models selecting a particular variable should tend to increase in direct proportion to the biologic relevance of the variable. There were 46 variables that were selected > 10,000 times in the 2,000-L10O process (Table 3), including all 12 *m*/*z* peaks identified by Qu et al. (2002) using boosted decision stump feature selection based on a slightly larger data set. The two-group *t*-test results indicated that 32 of 46 high-frequency variables have *p*-values < 0.001 (Table 3). Selection of 23 variables from Table 3 that were used in both fitting and L10O with *p* < 0.001 appears a reasonable approach to choosing a set of proteins for biomarker identification.

## Discussion

We developed a classification model for early detection of PCA on the basis of SELDI-TOF MS data using DF. DF is an ensemble method, where each prediction is a mean value of all the DT models combined to construct the DF model. The idea of combining multiple DT models implicitly assumes that a single DT model could not completely represent important functional relationships between predictor variables (*m*/*z* peaks in this study) and the associated outcome variables (PCA in this study), and thus different DT models are able to capture different aspects of the relationship for prediction. Given a certain degree of noise always present in omics data, optimizing a DT model inherently risks overfitting the noise. DF minimizes overfitting by maximizing the difference among individual DT models. The difference is achieved by constructing each individual DT model using a distinct set of predictor variables. Noise cancellation and corresponding signal enhancement are apparent when comparing the results from DF and DT. DF outperforms DT in all statistical measures in the 2,000 L10O runs. Whether DT performs better than other similar classification techniques depends on the application domain and the effectiveness of the particular implementation. However, Lim and Loh (1999) compared 22 DT methods with nine statistical algorithms and two artificial neural network approaches across 32 data sets and found no statistical difference among the methods evaluated. Thus, the better performance of DF than DT implies that the unique ensemble technique embedded in DF could also be superior to some other classification techniques for class prediction using omics data.

Combining multiple DT models to produce a single model has been investigated for many years (Bunn 1987, 1988; Clemen 1989; Zhang et al. 2003). Evaluating different ways for developing individual DT models to be combined has been a major focus, which have all been reported to improve ensemble predictive accuracy. One approach is to grow individual DT models based on different portions of samples randomly selected from the training set using resampling techniques. However, resampling using a substantial portion of samples (e.g., 90%) tends to result in individual DT models that are highly correlated, whereas using a less substantial portion of samples (e.g., 70%) tends to result in individual DT models of lower quality. Either high-correlated or lower-quality individual DT models can reduce the combining benefit that might otherwise be realized. The individual DT models can also be generated using more robust statistical resampling approaches such as bagging (Breiman 1996) and boosting (Freund and Schapire 1996). However, it is understood that boosting that uses a function of performance to weight incorrect predictions is inherently at risk of overfitting the noise associated with the data, which could result in a worse prediction from an ensemble model (Freund and Schapire 1996). Another approach to choosing an ensemble of DT models centers on random selection of predictor variables (Amit and Geman 1997). One popular algorithm, random forests, has been demonstrated to be more robust than a boosting method (Breiman 1999). However, in an example of classification of naive *in vitro* drug treatment sample based on gene expression data, Gunther et al. (2003) showed reduced prediction accuracy of random forests (83.3%) compared with DT (88.9%).

It is important to note that the aforementioned techniques rely on random selection of either samples or predictor variables to generate individual DT models. In each repeat the individual DT models of the ensemble are different; thus, the biologic interpretation of the ensemble is not straightforward. Furthermore, these methods need to grow a large number of individual DT models (> 400) and could be computationally expensive. In contrast the difference in individual DT models is maximized in DF such that a best ensemble is usually realized by combining only a few DT models (i.e., four or five). Importantly, because DF is reproducible, the variable relationships are constant in their interpretability for biologic relevance.

Omics data such as we stress in this article normally have a limited number of samples and a large number of predictor variables. Furthermore, the noise associating with both categorical dependent variables and predictor variables is usually unknown. It is consequently imperative to verify that the fitted model is not a chance correlation. To assess the degree of chance correlation of the PCA model, we computed a null distribution of prediction with 2,000 L10O runs based on 2,000 pseudo-data sets derived from a randomization test. The null hypothesis was tested by comparing the null distribution with the DF predictions in 2,000 L10O runs using the actual training data set. The degree of chance correlation in the predictive model can be estimated from the overlap of the two distributions (Figure 2). Generally speaking, a data set with an unbalanced sample population, small sample size, and/or low signal:noise ratio would tend to produce a model with distribution overlapping the null distribution. For the PCA model, the distributions are spaced far apart with no overlap, indicating that the model is biologically relevant.

A model fitted to omics data has minimal utility unless it can be generalized to predict unknown samples. The ability to generalize the model is an essential requirement for diagnostics and prognostics in medical settings and/or risk assessment in regulation. Commonly, test samples are used to verify the performance of a fitted model. Such external validation, while providing a sense of real-world application, must incorporate assurance that samples set aside for validation are representative. Setting aside only a small number of samples might not provide the ability to fully assess the predictivity of a fitted model, which in turn could result in the loss of valuable additional data that might improve the model. Besides, one rarely enjoys the luxury of setting aside a sufficient number of samples for use in external validation in omics research because in most cases data sets contain barely enough samples to create a statistically robust model in the first place. Therefore, an extensive L10O procedure is embedded in DF that can provide an unbiased and rigorous way to assess the fitted model’s predictivity within the available samples’ domain without the loss of samples set aside for a test set.

A model’s ability to predict unknown sample’s is directly dependent on the nature of the training set. In other words, predictive accuracy for different unknown samples varies according to how well the training set represents the given samples. Therefore, it is critical to be able to estimate the degree of confidence for each prediction, which could be difficult to derive from the external validation. In DF the information derived from the extensive L10O process permits assessment of the confidence level for each prediction. For the PCA model the confidence level for predicting unknown samples was assessed based on the distribution of accuracy over the prediction probability range for the left-out samples in the 2,000 L10O runs. We found that the sensitivity and specificity of the model were 99.2 and 98.2% in the HC region, respectively, with an overall concordance of 98.7%. In contrast, a much lower prediction confidence of 78.9% was obtained in the LC region, indicating that these predictions need to be further verified by additional methods. Generally, the number of samples within the HC region compared with the LC region depends on the signal:noise ratio in the data set. For noisy data, more unknown samples will be predicted in the LC region and could be as high as 40–50% (results not shown). For the PCA data set some 80% of the left-out samples predicted in the 2,000 L10O runs were in the HC region, indicating that the data set has a high signal:noise ratio.

A number of classification methods reported in the literature require selection of the relevant or informative predictor variables before modeling is actually performed. This is necessary because the method could be susceptible to noise without this procedure, and the computational cost is prohibitive for iterative variable selection during cross-validation. Although these are otherwise effective methods, they could produce what is called “selection bias” (Simon et al. 2003). Selection bias occurs when the model’s predictive performance is assessed using cross-validation where only the preselected variables are included. Because of selection bias, cross-validation could significantly overstate prediction accuracy (Ambroise and McLachlan 2002), and external validation becomes mandatory to assess a model’s predictivity. In contrast, model development and variable selection are integral in DF. DF avoids the selection bias during cross-validation because the model is developed at each repeat by selecting the variables from the entire set of predictor variables. The cross-validation thereby provides a realistic assessment of the predictivity of a fitted model. Given the trend of ever decreasing computation expense, carrying out exhaustive cross-validation is increasingly attractive, particularly when scarce sample data can be used for training as opposed to external testing. Of course, external validation is still strongly recommended when the amount of data suffices, in which case the cross-validation process will still enhance the rigor of the validation.

## Figures and Tables

**Table 1:** Summary of the four DT models combined for developing the DF model (*n* = number of misclassifications).

	DT model 1 (*n* = 12)	DT model 2 (*n* = 13)	DT model 3 (*n* = 14)	DT model 4 (*n* = 14)
Variables (*m*/*z* peaks) used in each DT model	9,656	8,067	6,542	7,692
	8,446	8,356	7,934	6,756
	5,074	5,457	7,195	9,593
	6,797	2,144	4,497	9,456
	8,291	7,885	4,080	5,978
	9,720	7,024	6,199	3,780
	3,486	7,771	7,481	2,794
	4,191	3,897	5,586	7,844
	4,653	4,757	6,099	5,113
		6,890	7,070	28,143
		2,014	24,400	2,982
		9,149	2,887	6,443
			7,054	7,820
			4,475	4,580
			4,537	
			7,409	
			7,054	

**Table 2:** Comparison of statistics between DF and DT models in prediction of the left-out samples in the 2,000 L10O runs.

Prediction accuracy	DF (%)	DT (%)
Overall accuracy	94.7	89.4
Accuracy in HC region	98.7	90.7
Accuracy in LC region	78.9	63.8

**Table 3:** List of *m*/*z* peaks used more than 10,000 times in the 2,000-L10O process, where 23 peaks are used in fitting with *p* < 0.001.

*m*/*z* Peaks (Da)	Frequency	*p*-Value
7,934*^a^*	30,203	< 0.001
9,149*^a^*	26,482	< 0.001
7,984*^b^*	25,171	< 0.001
8,296*^a^*	24,793	< 0.001
3,897*^a^*	23,754	< 0.001
9,720^a,c^	22,630	< 0.001
7,776*^a^*	21,723	0.003
7,024^a,c^	21,718	< 0.001
5,074*^a^*	20,800	< 0.001
8,446*^a^*	20,620	< 0.001
9,656^a,c^	20,479	< 0.001
6,542^a,c^	20,219	< 0.001
8,067^a,c^	20,058	< 0.001
7,692*^a^*	19,982	0.004
6,797^a,c^	19,587	< 0.001
8,356^a,c^	19,429	< 0.001
7,054*^a^*	19,333	0.010
6,099*^a^*	19,265	0.004
5,586*^a^*	18,103	< 0.001
7,820^a,c^	17,918	0.359
6,756*^a^*	17,668	< 0.001
9,593*^a^*	17,615	< 0.001
7,844*^a^*	17,611	0.089
4,191*^a^*	17,387	< 0.001
3,486*^a^*	17,290	< 0.001
4,451*^b^*	17,041	0.459
4,079^a,c^	16,790	0.020
9,456*^a^*	16,767	< 0.001
4,653*^a^*	16,674	0.002
7,195*^a^*	15,832	< 0.001
7,885^a,c^	15,388	< 0.001
8,277*^b^*	15,388	< 0.001
6,072*^b^*	15,093	< 0.001
3,963^b,c^	14,434	< 0.001
3,780*^a^*	14,139	0.014
4,291*^b^*	13,540	< 0.001
4,102*^b^*	13,294	0.001
4,858*^b^*	13,076	0.003
6,949^b,c^	12,555	< 0.001
3,280*^b^*	11,808	< 0.001
6,991^b,c^	11,281	0.122
2,144*^a^*	11,110	< 0.001
9,100*^b^*	10,578	< 0.001
7,652*^b^*	10,159	0.005
5,457*^a^*	10,139	< 0.001
6,914*^b^*	10,073	< 0.001

a Used in fitting.

b Not used in fitting.

c Reported by Qu et al. (2002).
